# Partners in crime: neutrophils and monocytes/macrophages in inflammation and disease

**DOI:** 10.1007/s00441-017-2753-2

**Published:** 2018-01-31

**Authors:** Kathryn Prame Kumar, Alyce J. Nicholls, Connie H. Y. Wong

**Affiliations:** Centre for Inflammatory Diseases, Department of Medicine, School of Clinical Sciences, Monash Medical Centre, Monash University, Clayton, VIC 3168 Australia

**Keywords:** Inflammation, Neutrophils, Monocytes/macrophages, Atherosclerosis, Glomerulonephritis, Inflammatory bowel disease

## Abstract

Neutrophils are becoming recognized as highly versatile and sophisticated cells that display de novo synthetic capacity and potentially prolonged lifespan. Emerging concepts such as neutrophil heterogeneity and plasticity have revealed that, under pathological conditions, neutrophils may differentiate into discrete subsets defined by distinct phenotypic and functional characteristics. Indeed, these newly described neutrophil subsets will undoubtedly add to the already complex interactions between neutrophils and other immune cell types for an effective immune response. The interactions between neutrophils and monocytes/macrophages enable the host to efficiently defend against and eliminate foreign pathogens. However, it is also becoming increasingly clear that these interactions can be detrimental to the host if not tightly regulated. In this review, we will explore the functional cooperation of neutrophil and monocytes/macrophages in homeostasis, during acute inflammation and in various disease settings. We will discuss this in the context of cardiovascular disease in the form of atherosclerosis, an autoimmune disease mainly occurring in the kidneys, as well as the unique intestinal immune response of the gut that does not conform to the norms of the typical immune system.

## Introduction

The earliest reference to the concept of “immunity” was made in Athens in 430 BC. Thucydides, an Athenian historian, reported that individuals who had previously recovered from the plague were able to tend to the sick without relapsing (Wylie and Stubbs 2009). Since then, numerous studies have highlighted the complexity and importance of the immune system. The innate immune system is the first line of defense against invading pathogens comprising various mechanisms from physical barriers to cellular components (Alberts et al. 2002). Upon recognition of a pathogen, the innate immune system mounts a broad immune response to abate infection and, in most cases, the adaptive immune system is instrumental to confer longer-lasting protection. Unlike the adaptive immunity, the innate immune system is not confined to vertebrates as it is present in all types of plants and animals (Janeway et al. 2001). Indeed, to be able to defend against the dynamic and ever-changing microenvironment populated by numerous and potentially infectious microbial communities surrounding us, various members of the immune system interact to create and maintain a tailored immune response. One such interaction explored here is between neutrophils and monocytes/macrophages.

Polymorphonuclear leukocytes, more commonly known as neutrophils, along with monocytes/macrophages arise from common precursors and, due to this, it is expected that they share common features (McCracken and Allen 2014; Silva 2010). In fact, these cells are essential professional phagocytes that are capable of carrying out various roles in the host’s innate defense against pathogens (Butterfield et al. 2006). In addition, neutrophils and monocytes/macrophages co-express similar antigens and these innate phagocytes can readily produce effector molecules such as granular proteins, oxidants, chemokines and cytokines (Daley et al. 2008; Nauseef 2007; Sunderkötter et al. 2004). Regardless of their similarities, emerging evidence indicates that neutrophils and monocytes/macrophages have distinct roles as innate immune cells and therefore are indispensable as key players against infection. Typically, neutrophils are the first responders to be recruited and have a higher microbicidal activity; whereas monocytes/macrophages are recruited later on. Despite this, monocytes/macrophages are able to digest and present antigens to other immune cells, thereby allowing them to interact with the adaptive immune system (Silva and Correia-Neves 2012). Neutrophils and monocytes/macrophages share a complex relationship and; together, they orchestrate a more enhanced immune response by regulating other immune cells as well as each other.

### Neutrophils and monocyte/macrophages coordinate an effective immune response

**Fig. 1:**
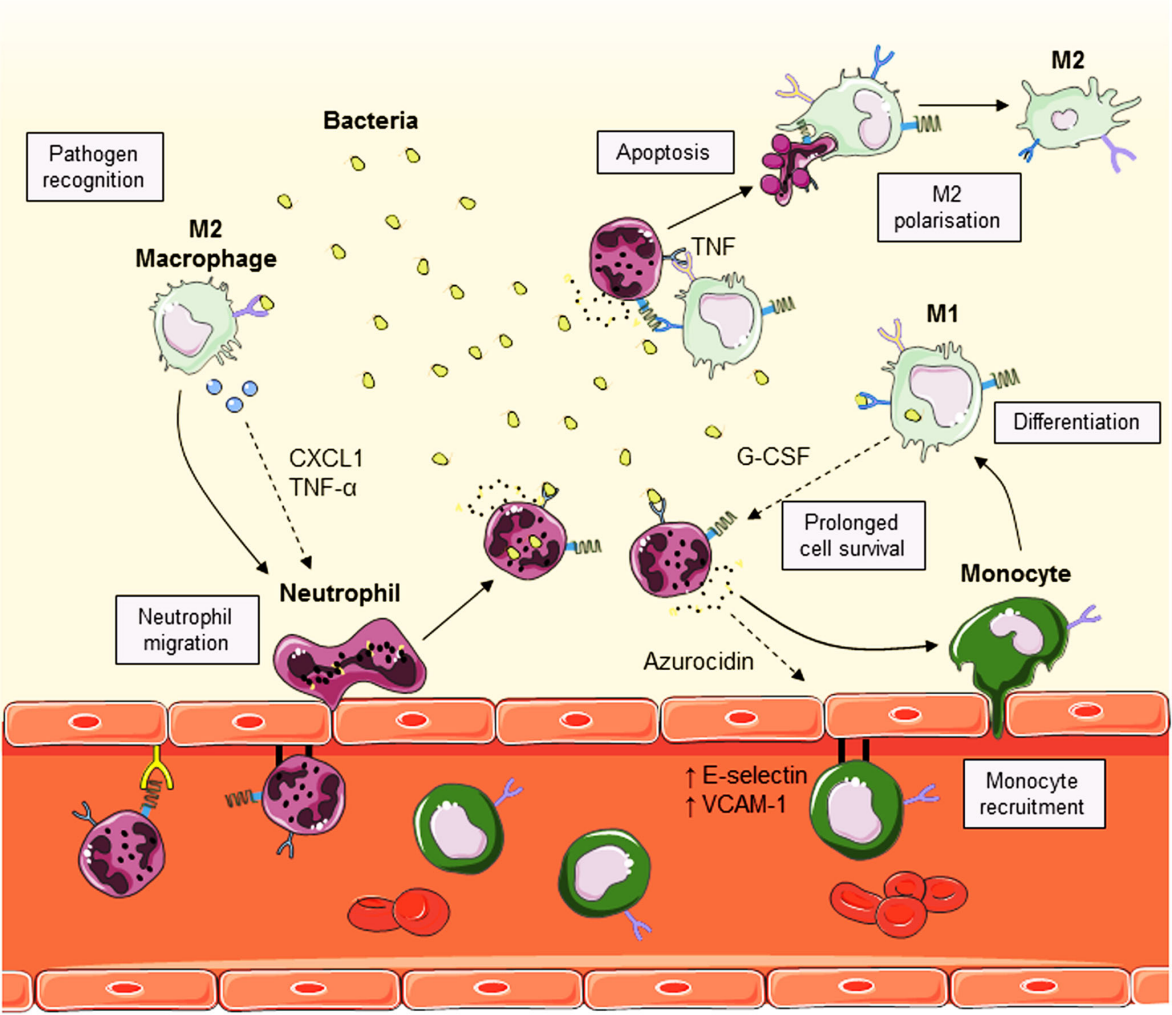
Neutrophil–macrophage interaction during an immune response. Following pathogen recognition, tissue-resident M2 macrophages produce CXCL1 and tumor necrosis factor-alpha (*TNF-α*), which are involved in neutrophil recruitment and granulopoiesis. The recruited neutrophils destroy the invading pathogens and produce azurocidin that upregulates E-selectin and vascular cell adhesion molecule-1 (*VCAM-1*) expression on the endothelium to enhance monocyte recruitment. Monocytes that have transmigrated into the tissues differentiate into M1 macrophages and proceed to degrade the invading pathogens and produce granulocyte colony-stimulating factor (*G-CSF*) to prolong neutrophil survival. Once inflammation is resolved, M1 macrophages bind to neutrophils via TNF to induce apoptosis. Apoptotic neutrophils are cleared away by M1 macrophages that then polarize toward an M2 phenotype to restore homeostasis. (Stock images sourced from Servier Medical Art; Creative Commons)

Due to their robust reactivity to pathogens, neutrophils are typically not resident in body cavities. Instead, neutrophils are produced and stored in large reserves in the bone marrow, ready to be deployed into the circulation (Yamashiro et al. 2001). In fact, neutrophils are the most abundant circulating leukocyte in humans and are equipped with potent microbicidal activity (McCracken and Allen 2014). Monocytes also originate from the bone marrow but circulating monocytes can give rise to macrophages and dendritic cells (DC) (Geissmann et al. 2010). In contrast to neutrophils, tissue-resident macrophages are less immunoreactive; which is an important feature that enables them to patrol the tissues for pathogens (Davies and Taylor 2015). Following microbial challenge, tissue-resident macrophages become activated to produce neutrophil chemoattractants such as CXCL1, CXCL2, interleukin (IL)-1α and monocyte chemoattractant protein-1 (MCP-1) (Fig. 1) (Barry et al. 2013; Beck-Schimmer et al. 2005; De Filippo et al. 2008). The resultant effect is a rapid influx of neutrophils to the site of infection. However, it is traditionally thought that the lifespan of recruited neutrophils is relatively short as they are preprogrammed to die quickly to prevent excessive inflammation (McCracken and Allen 2014). Therefore, macrophages act to prolong their survival by producing a variety of growth factors such as granulocyte–macrophage colony-stimulating factor (GM-CSF), granulocyte colony-stimulating factor (G-CSF) and tumor necrosis factor alpha (TNF-α) (Takano et al. 2009). This event typically marks the onset of inflammation. Evidently, neutrophils and macrophages work in concert to enhance the immune response against pathogens; however, it is important that this relationship is tightly regulated as it may contribute to overt inflammation and onset of pathology.

Once recruited, neutrophils are capable of inducing a second-wave inflammatory response. Firstly, the dynamic neutrophil phenotype is altered to suit the changing environment (Rao et al. 2014). As this occurs, neutrophils regulate the release of chemoattracting factors, such as cathepsin G and azurocidin, which are involved in the recruitment of other immune cells, in particular monocytes/macrophages (Fig. 1) (Chertov et al. 1997a). These cells initiate a feed-forward loop that involves further downstream inflammatory processes (Hu et al. 2008). In addition, neutrophil-derived azurocidin was shown to attract inflammatory monocytes in vivo (Soehnlein et al. 2008c). The underlying mechanism was shown to involve the upregulation of various adhesion molecules on the endothelium, including intercellular adhesion molecule-1 (ICAM-1), vascular cell adhesion molecule-1 (VCAM-1) and E-selectin (Lee et al. 2003). Moreover, neutrophils are able to alter vascular permeability by inducing changes in the cytoskeletal structure of endothelial cells, thus promoting the transmigration of monocytes (Gautam et al. 2001). Studies involving neutrophil depletion have highlighted the importance of neutrophil influx for subsequent monocyte/macrophage recruitment. In fact, mice depleted of neutrophils demonstrated a decrease in mononuclear inflammatory cell infiltrate, specifically inflammatory monocytes (Soehnlein et al. 2008c; Zhou et al. 2003). Conversely, reconstitution with neutrophils restored this loss of inflammatory monocytes recruitment (Soehnlein et al. 2008c). In a mouse model of neutrophil-specific granule deficiency, the distribution of cell-surface markers on monocytes was lowered (Shiohara et al. 2004). Moreover, lipopolysaccharide (LPS) stimulation of monocytes isolated from the peripheral blood of patients with neutropenia was found to have reduced cytokine production compared to healthy controls (Mokart et al. 2008). Collectively, these studies demonstrate that neutrophils are essential for monocyte/macrophage recruitment and function.

In a classical immune response, the granular products released by neutrophils directly act on pathogens with potent antimicrobial activity. It has become apparent that these proteins are also able to affect the activity of neighboring monocytes and macrophages. Depending on the microenvironment they find themselves in, macrophages are able to acquire either the “M1” or “M2” phenotype, hence allowing the macrophage to act in a pro-inflammatory or anti-inflammatory manner, respectively (Hamilton et al. 2014). The M1/M2 paradigm is gaining increasing recognition in the field but it should be noted that it is a gliding scale and that a clear M1/M2 distinction is oversimplified. Pro-inflammatory macrophages express inducible nitric oxide synthase and CD40 and produce TNF-α and IL-6, whereas anti-inflammatory macrophages express arginase I and CD206 and produce transforming growth factor (TGF)-β and IL-10 to facilitate tissue repair (Liu et al. 2013; Wynn and Vannella 2016). During an infection, neutrophils typically induce a M1 phenotype in macrophages to prime their pro-inflammatory activity. One of the mechanisms by which neutrophils mediate macrophage polarization is by their release of azurocidin (Fig. 1) (Påhlman et al. 2006). Lactoferrin, another neutrophil granule product, was detected within peritoneal macrophages upon infection with *Mycobacteria*. This protein is not synthesized by macrophages and therefore it is thought to be transferred from apoptotic neutrophils during their clearance by macrophages. Not surprisingly, the antimicrobial activity of macrophages containing lactoferrin was significantly enhanced following infection (Silva et al. 1989). This finding was corroborated by a study that specifically tracked the uptake of neutrophil-derived granules by macrophages. Following this acquisition, the macrophages showed increased microbicidal activity (Tan et al. 2006). Furthermore, a recent study reported that IL-13 secreted by neutrophils is able to skew macrophage polarization to the M2 phenotype during a helminth infection to effectively eradicate these parasitic worms (Chen et al. 2014). These findings highlight the versatility of neutrophils as they are able to both recruit and augment the activity of macrophages according to the demands placed upon them by the immune system.

There are various homeostatic mechanisms put in place by the immune system to prevent an accumulation of leukocytes in the tissue and thus avoid overt inflammation. Apoptosis, also known as programmed cell death, is a biological response that occurs following the resolution of inflammation (Elmore 2007). Macrophages possess membrane-bound TNF allowing them to induce neutrophil cell death (Allenbach et al. 2006). These apoptotic neutrophils are then cleared away by tissue-resident macrophages via phagocytosis (Poon et al. 2014). Annexin A1 (AnxA1), a protein produced by neutrophils, enhances this process as it increases the phagocytic ability of macrophages (Scannell et al. 2007). It was found that phagocytosis of apoptotic neutrophils causes the macrophage to acquire a M2 phenotype (Fig. 1). In these macrophages, the production of inflammatory mediators such as IL-23, a cytokine involved in granulopoiesis, is inhibited, while the secretion of TGF-β1, an important regulatory cytokine, is increased (Fadok et al. 1998; Stark et al. 2005). In addition, AnxA1 is able to act as a negative regulator of neutrophil accumulation as it was shown to be a monocyte chemoattractant. To expand, the monocytes recruited by AnxA1 differentiate into macrophages, which then proceed to engulf and clear away the remaining neutrophils in the tissue (Chertov et al. 1997a). Senescent monocytes and macrophages themselves undergo apoptosis and are cleared away by scavengers (Poon et al. 2014). Taken together, there are various mechanisms utilized by neutrophils and monocytes/macrophages to regulate the clearance of cells in the resolution of inflammation.

There is also emerging evidence suggesting that neutrophils are able to take on different phenotypes and functions during physiological conditions as well as in various diseases such as diabetes, stroke and myocardial infarction (Cuartero et al. 2013; Ma et al. 2016; Rao et al. 2014). Perhaps non-intuitively, neutrophils in the skin have been shown to be critical for the resolution of tissue inflammation by facilitating wound healing (Cantürk et al. 2001). Therefore, it is becoming clear that simply removing neutrophils or halting their recruitment following inflammation has the potential to impact on inflammatory responses in a more complex fashion than originally believed. In contrast to previous dogma, neutrophils are becoming recognized as highly versatile and sophisticated cells that display de novo synthetic capacity and potentially prolonged lifespan (de Oliveira et al. 2016; Mantovani et al. 2011). In addition, concepts such as “neutrophil heterogeneity” and “neutrophil plasticity” have begun to emerge, with evidence indicating that, under pathological conditions, neutrophils may differentiate into discrete subsets defined by distinct phenotypic and functional characteristics (Beyrau et al. 2012). Indeed, it was reported that neutrophils acquire the “N2” immunosuppressive phenotype during tumourigenesis and chronic inflammation (de Oliveira et al. 2016). These N2 neutrophils are characterized by their immunosuppressive ability and delay in apoptosis as there is a reduction in Fas-ligand expression (Andzinski et al. 2015). Additionally, classical neutrophil function was attenuated in the N2 neutrophils as there was a downregulation in the pathways associated with antigen processing and chemokines (Shaul et al. 2016). TGF-β blockade resulted in the decrease of N2 neutrophils suggesting that this cytokine is important in inducing an immunosuppressive profile in neutrophils (Fridlender et al. 2009). Interestingly, M2 macrophages in the intestines are producers of TGF-β and Fas-ligand and therefore there is ongoing research to examine whether the change in the neutrophil phenotype may also occur in the host tissues of various immunological microenvironments and whether this is dependent on the tissue-resident macrophages.

Overall, the interactions between neutrophils and monocytes/macrophages enable the host to effectively defend against and eliminate foreign pathogens; however, it is also becoming clear that these interactions can be detrimental to the host if not tightly regulated. The latter part of this review explores the functional cooperation of neutrophils and monocytes/macrophages in various disease settings. We will discuss these interactions in the context of cardiovascular disease in the form of atherosclerosis, an autoimmune disease mainly occurring in the kidneys, as well as the unique intestinal immune response of the gut that does not conform to the norms of the typical immune system.

### Neutrophils and monocyte/macrophages in atherosclerosis

Atherosclerosis, a disease of the vasculature, is characterized by progressive accumulation of low-density lipoproteins (LDLs), fibrosis and inflammation within the vascular endothelium. Development of fatty plaques as a result of these processes leads to narrowing of the vascular lumen and is associated with myocardial infarction and stroke (Bots et al. 1997; Grau et al. 2001; O’Leary et al. 1999). For many years, the development and progression of atherosclerotic plaques have traditionally been viewed as a classical monocyte-driven process (Gerrity, 1981; Østerud and Bjørklid 2003; Woollard and Geissmann 2010). Once activation of the vascular endothelium is triggered, interaction between monocytes and the endothelium, mediated by adhesion molecules, induces monocyte arrest and extravasation into the endothelial space (Mestas and Ley 2008). Here, the cells differentiate into macrophages producing pro-inflammatory cytokines and reactive oxygen species (ROS) and are capable of ingesting vast amounts of accumulated oxidized LDLs (Weber and Noels, 2011). Along with pro-inflammatory macrophages within the fatty streak, foam cell death propagates inflammation to perpetuate the development of the plaque (Soehnlein and Weber, 2009).

It is well accepted that hyperlipidemia increases the risk for developing atherosclerosis by way of increasing the number of circulating inflammatory monocytes and their emigration into atherosclerotic lesions (Adamson and Leitinger 2011; Huang et al. 2001; Mohty et al. 2008; Shankar et al. 2007). However, emerging evidence is accumulating that hyperlipidemia not only activates monocytic cells but also induces neutrophilia and priming of circulating neutrophils (Drechsler et al. 2010; Huang et al. 2001). With the advancement of neutrophil-specific fluorescent labeling techniques in recent years, a number of studies have demonstrated the accumulation of neutrophils within atherosclerotic plaques (Ionita et al. 2010; Rotzius et al. 2010). In fact, hyperlipidemia- or hypercholesterolemia-induced neutrophilia has been shown to be an initiating stimulus for plaque development (Drechsler et al. 2010). While the total number of neutrophils within the plaque is minimal, the locations of maximal neutrophil infiltrations correlate with those regions having the highest monocyte density (Rotzius et al. 2010). To examine the role of neutrophils in atherosclerosis, it was found that the administration of a CXCR4 antagonist to induce neutrophilia in Apolipoprotein E knockout (*Apoe*^*−/−*^) mice triggered the rapid development of atherosclerosis due to abnormal lipoprotein metabolism (Zernecke et al. 2008). These mice exhibited enhanced lesion formation with a modest increase in lesion monocyte numbers compared to mice that did not receive the CXCR4 antagonist. Conversely, prevention of neutrophil trafficking to sites of inflammation through adoptive transfer of neutrophils deficient in the chemokine receptor CXCR2 was found to be protective against atherosclerotic plaque formation (Zernecke et al. 2008). In a separate study, induction of neutropenia in mice significantly reduced the number of monocytes and macrophages within atherosclerotic plaques and attenuated plaque development (Drechsler et al. 2010). Furthermore, antagonism of the receptor for a potent neutrophil chemokine, IL-8 receptor, was also shown to be effective in the attenuation of atherosclerosis (Qin et al. 2013). Neutrophils may play direct roles in lesion formation, for example, through modification of LDLs by myeloperoxidase (MPO), making them increasingly recognizable to macrophages and making high-density lipoproteins no longer able to remove cholesterol from foam cells (Podrez et al. 1999; Undurti et al. 2009). However, their low numbers and co-localization with monocytes within plague regions vulnerable to rupture, point to a role for leukocyte interactions in atherogenesis (Ionita et al. 2010; Rotzius et al. 2010). Much research has thus been undertaken to understand the mechanisms by which neutrophil–monocyte interactions contribute to lesion formation (Ionita et al. 2010; Soehnlein et al. 2005).

**Fig. 2:**
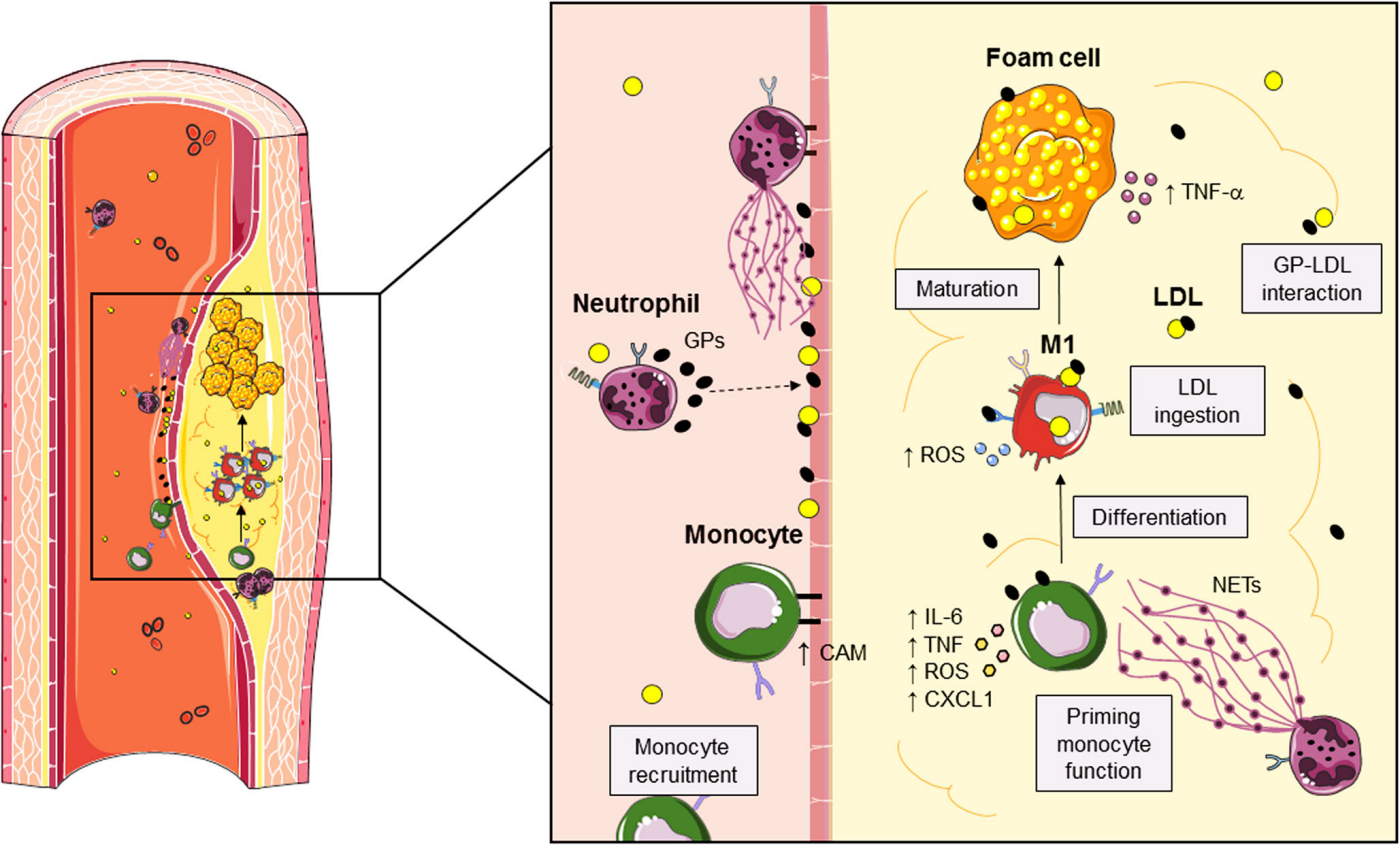
Immune interactions in atherosclerotic plaques. During atherosclerosis, neutrophils release granule proteins (*GPs*) that are deposited along the endothelial vessel to regulate the expression of cellular adhesion molecule (*CAM*). Monocytes traveling into the plaque differentiate into M1 macrophages and ingest surrounding LDLs causing their maturation to foam cells. Within the atherosclerotic plaques, GPs released from intralesional neutrophils interact with low-density lipoproteins (*LDL*), making them more recognizable to macrophages. GPs can also interact with monocytes, macrophages and foam cells to increase their proinflammatory, phagocytic activity. Concurrently, neutrophils can release their nuclear contents in the form of extracellular traps (*NETs*) to prime monocyte function causing the release of CXCL1 from monocytes, resulting in a positive feedback loop to enhance neutrophil recruitment. *ROS* reactive oxygen species; *TNF* tumor necrosis factor. (Stock images sourced from Servier Medical Art; Creative Commons)

One such mechanism is the neutrophil-driven recruitment of monocytes to sites of lipid accumulation (Soehnlein et al. 2008c). Exocytotic release of granules containing pro-inflammatory peptides is thought to be a major mechanism by which neutrophils perform these functions (Fig. 2) (Soehnlein et al. 2005). In vivo models have demonstrated their importance by depleting neutrophils in mice and subsequently superfusing neutrophil secretions onto the tissue. Neutropenic mice exhibited a marked reduction in the accumulation of monocytes at a site of inflammation that can be reversed by the subsequent exposure to granule products (Soehnlein et al. 2008c). In human atherosclerotic plaques, the use of immunohistochemistry has provided evidence for LL-37 deposition within the neointima, while cathepsin G has been established by the presence of its mRNA (Ciornei et al. 2006; Legedz et al. 2004). Additional granule products, including azurocidin and α-defensins have also been identified (Barnathan et al. 1997; Lee et al. 2002). These granule products have been shown to be chemotactic for monocytes by several mechanisms, including Ca^2+^ mobilization by FPRL1 activation and modification of macrophage inflammatory protein (Agerberth et al. 2000; Chertov et al. 1997b; Soehnlein et al. 2009b; Yang et al. 2000). Azurocidin deposition on the endothelial surface, triggered by neutrophil–integrin binding, led to monocyte adhesion and extravasation into the tissue (Lee et al. 2003), whereas another study demonstrated that release of azurocidin onto the vascular endothelium stimulated arrest of monocytes, potentially occurring through the priming effect of CAM on monocyte rolling prior to adhesion (Fig. 2) (Soehnlein et al. 2005).

Once at the site of lipid accumulation, the function of monocytes/macrophages and foam cells can also be modulated by granule products released by neutrophils. In vitro studies have demonstrated that culturing monocytes with LL-37 shapes their development into a pro-phagocytic, M1 phenotype, while culture of LL-37 with fully differentiated macrophages can shift them away from an M2 phenotype. (Soehnlein et al. 2008c; van der Does et al. 2010; Wan et al. 2014). This priming in the phagocytic capacity of granule-peptide-treated macrophages may occur through increased generation of ROS (Fig. 2) (Soehnlein et al. 2008b). α-defensins also activate macrophages to enhance phagocytosis through the generation of ROS in addition to causing an upregulation in cytokine synthesis (Soehnlein et al. 2008a; Zughaier et al. 2005). A lack of interaction between monocytes and neutrophil granule products via neutropenia correlates with decreased circulating levels of IL-6 and TNF (Lee et al. 2003). Notably, the levels of these primarily monocyte-derived cytokines can be re-established by administration of granule products (Soehnlein et al. 2008c). Neutrophil granule products can also regulate development and function of foam cells. For example, foam cell maturation was shown to be impaired by blockade of the FPR2 receptor, which can recognize LL-37 as an agonist and this correlated with reduced production of TNF-α (Lee et al. 2013, 2014).

In addition to modulation via the controlled release of granule products, neutrophil extracellular trap (NET) formation is capable of priming monocyte function in atherosclerosis (Fig. 2) (Fuchs et al. 2007; Nahrendorf and Swirski 2015). NETs are web-like structures, consisting of neutrophil DNA and endogenous antimicrobial proteins, extruded upon stimulation leading to death of the cell (Remijsen et al. 2011). Increased numbers of netting neutrophils have been found adhering to the vascular lumen in mouse models of atherosclerosis, as well as being identified in atherosclerotic plaques removed from human arteries (Megens et al. 2012; Warnatsch et al. 2015). To examine the effects of NET formation by cholesterol crystal-primed neutrophils on atherogenesis, *Apoe*^*−/−*^ mice unable to undergo NET formation fed on high fat had a 3-fold decrease in the size of atherosclerotic lesions and this finding was similarly observed by other researchers (Knight et al. 2014; Warnatsch et al. 2015). Furthermore, while NETs were found to be a crucial driver in the synthesis of pro-inflammatory, pro-atherogenic cytokines, such as IL-1β and IL-6, these cytokines were not neutrophil-derived. Instead, monocytes exposed to NETs and subsequently to cholesterol crystals, exhibited a substantial increase in their production of these cytokines. Furthermore, chemokines such as CXCL1 and CXCL2 released by these primed monocytes then contributed to a positive feedback loop by further attracting neutrophils to the site of lipid and monocyte accumulation (Fig. 2).

It is becoming increasingly clear that phagocyte partnership, through cell mediators, is a key aspect in the development of atherosclerosis. Disruption in the recruitment or function of either one of the cell types discussed can disrupt this partnership and afford considerable protection against plaque formation and the risk of the associated complications. In future, taking advantage of our understanding in neutrophil–monocyte cell interactions may help in the development of therapeutics to aid in the prevention of atherogenesis.

### Neutrophils and monocytes/macrophages in the kidney

Glomerulonephritis (GN) is a global term referring to a collection of kidney diseases mostly involving inflammation of the glomeruli or renal vasculature. These can be acute or chronic in nature, can be generally classified as non-proliferative or proliferative and can present as a nephrotic or nephritic syndrome. Common clinical characteristics of these diseases can include hematuria, proteinuria, hypertension, edema and decreased urine output (Becquet et al. 2010; Hamouda et al. 2014; Li et al. 2014; Ramanathan et al. 2017). GN is commonly attributed to non-infectious causes including immune complex-mediated disease, or as a complication arising from cardiac surgery or critical illness requiring intensive care, although post-infectious causes also exist (Jegatheesan et al. 2016; Kanjanabuch et al. 2009; Li et al. 2011; Okpechi et al. 2010; Rosner and Okusa 2006). Kidney damage during GN, such as an acute kidney injury (AKI), is known to result from a multitude of factors including activation of the complement cascade, endothelial damage, leukocyte infiltration, pro-inflammatory signaling, necrosis and apoptosis (Akcay et al. 2010; Allam et al. 2012; Kaushal et al. 2004; Thurman et al. 2003). Although GN patients may appear to recover from an episode of AKI, with serum creatinine returning to normal levels, alterations in kidney physiology mean that AKI is a known predictor of chronic kidney disease and end-stage renal disease (Lech et al. 2014). In patients undergoing cardiac surgery who develop AKI, the risk of mortality is high and correlates with the severity of injury (Abel et al. 1976; Lech et al. 2014; Rosner and Okusa 2006). More specifically, research has demonstrated that the immune response associated with an episode of AKI is a major determinant of patient outcomes (Zhang et al. 2015).

Many immune cell types have been implicated in the development of proliferative GN; however, phagocytic leukocytes have been shown to be major contributors in the pathogenesis of kidney injury, particularly in crescentic and post-infectious GN (Ferrario et al. 1985; Hooke et al. 1987). In fact, monocytes/macrophages appear to be the most abundant immune cell type within the glomerulus in several forms of GN (Hooke et al. 1987; Weidner et al. 2004). While leukocytes do not typically accumulate in capillary beds, their aberrant recruitment to the glomerular capillaries represents a major pathway for tissue injury (Braehler et al. 2016; Finsterbusch et al. 2016; Xiao et al. 2005). Within hours of the induction of injury, influx of inflammatory monocytes/macrophages and neutrophils contribute to early tubular necrosis through the generation of pro-inflammatory cytokines, myeloperoxidase and extracellular traps (Braehler et al. 2016; O’Sullivan et al. 2015). In particular, generation of ROS and secretion of proteolytic enzymes by neutrophils have long been known to significantly disrupt the integrity of the glomerular capillaries leading to glomerular dysfunction causing symptoms such as proteinuria (Baud and Ardaillou 1993; Odobasic et al. 2007). Neutrophils can also contribute to monocyte activation and extravasation through granule product release and extracellular trap formation (Nakazawa et al. 2016; Soehnlein et al. 2009a). In contrast, in the later stages of disease, M2-type macrophages have been shown to be important in inflammation resolution and tissue repair (Jang and Rabb 2009; Lee et al. 2011).

More recently, several key studies have highlighted not only the role of these phagocytes in modulating renal injury but also the importance of novel monocyte–neutrophil interactions in the propagation of inflammation, compared with other tissue compartments. Using a mouse model of renal ischaemia reperfusion injury (IRI), a major cause of sterile AKI, CD169^+^ kidney-resident monocytes and macrophages exert protective effects in the glomerulus through prevention of neutrophil accumulation (Karasawa et al. 2014). In this study, targeted depletion of CD169^+^ monocytes/macrophages resulted in significantly elevated and ongoing renal injury compared to that of their wild-type counterparts. These cells form a major compartment of the CX3CR1^+^ peripheral monocytes and macrophages residing in the kidney. Even more notably, depletion of CD169^+^ monocytes/macrophages prior to renal IRI proved to be fatal within 2 days of injury. Further investigation of these findings using flow cytometry and immunohistochemistry revealed that this effect was due to increased accumulation of neutrophils in the kidney. Additionally, the depletion of neutrophils in CD169-DTR mice attenuated the disease, highlighting the deleterious role of neutrophils in AKI. The protective effect afforded by CD169^+^ monocytes/macrophages was mediated by the suppression of ICAM-1 and macrophage inflammatory protein 2-alpha (MIP2α)/CXCL2 upregulation, potentially to inhibit further neutrophil or monocyte recruitment.

In a separate model of glomerular inflammation, monocytes and neutrophils were also found to be retained in the intravascular space following administration of an anti-glomerular basement membrane (GBM) antibody (Devi et al. 2013). In the uninflamed kidney, non-classical monocytes were observed to patrol the glomeruli of the kidney in addition to trafficking neutrophils. However, upon administration of the anti-GBM antibody, there was a significant increase in the duration of retention of monocytes and neutrophils within the intravascular space. This finding was not seen in a model of puromycin aminonucleoside nephrosis but increased neutrophil dwell time has been similarly observed in models using anti-MPO (Kuligowski et al. 2009). Intriguingly, this response appears to be unique to the kidney, with studies of inflammation in skeletal muscle not demonstrating marked increases in neutrophil dwell time.

In this model, a significant increase in oxidative burst was observed for those neutrophils that were retained longer in the glomerulus and inhibition of the macrophage-1 (MAC-1) antigen led to restoration of neutrophil behavior to basal levels. Importantly, diminution in the dwell time of, and oxidant production by, neutrophils was associated with a marked reduction in albuminuria, reflecting amelioration in disease pathology. Other studies have demonstrated that neutrophils persist in the glomerulus only for a short time frame following administration of anti-GBM (Tang et al. 1997). Despite this brief retention, ROS-producing neutrophils, along with monocytes, appear to be capable of inducing significant and enduring damage to the kidney and play a critical role in the pathogenesis of AKI.

Another study extended these findings by imaging direct interactions between intravascular neutrophils and monocytes within the glomerulus upon the initiation of glomerulonephritis (Finsterbusch et al. 2016). In the same model of anti-GBM-induced glomerulonephritis, it was reported that direct interactions between monocytes and neutrophils occur for increasing durations within the inflamed kidney (Finsterbusch et al. 2016). In fact, these interactions primed the cells for subsequent inflammatory activity. When neutrophils directly interacted with patrolling monocytes within the intravascular glomerulus, they were more likely to be activated and were retained for a longer duration of time than those that did not interact. Not only did these cells remain longer in the glomerulus but they were also observed to have increased production of ROS compared with non-interacting cells. To confirm these findings, mice depleted of monocytes also demonstrated a significant decrease in ROS production by neutrophils. In an effort to understand how this communication leads to activation of neutrophil ROS production, anti-TNF antibodies were administered. Upon inhibition, the dwell time of neutrophils in the glomerulus was reduced and there was a substantial decrease in the number of cells producing ROS, correlating with reduced glomerular injury. Taken together, these studies demonstrate that while neutrophils may only remain within the glomerular capillary for a short time frame, their recruitment and activation by monocytes is detrimental to glomerular structure and function. Disruption in the recruitment of neutrophils to the glomerulus or inhibiting the interaction between monocytes and neutrophils in the glomerulus may prove to be therapeutic for the treatment of GN. Furthermore, increased understanding of these novel leukocyte interactions in the kidney may shed light on the mechanisms of recruitment, activation and regulation of these cell types in other disease processes.

### Neutrophils and monocytes/macrophages in the gut

**Fig. 3:**
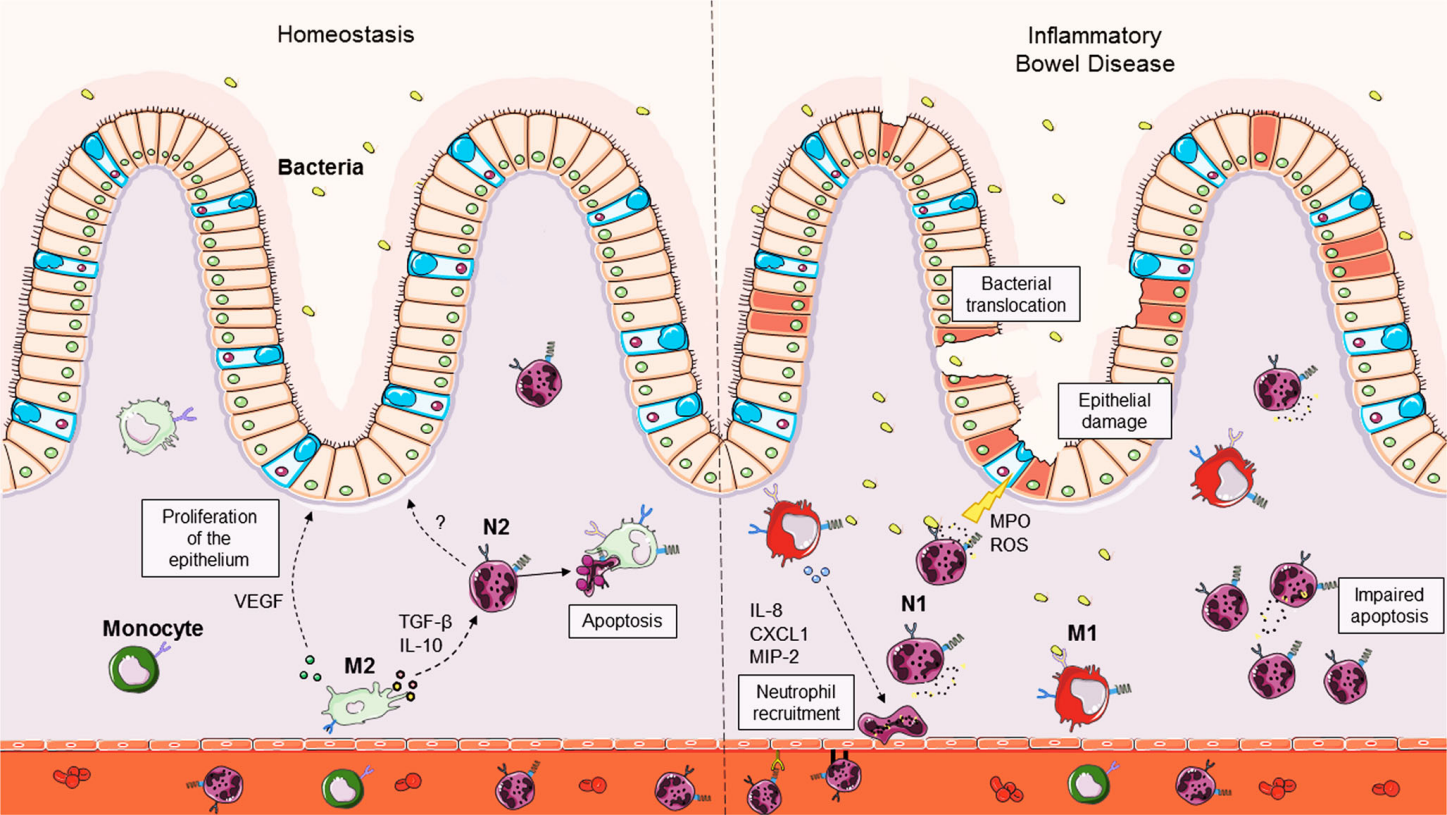
Intestinal homeostasis vs inflammatory bowel disease (IBD). During intestinal homeostasis, M2 macrophages mainly release anti-inflammatory factors that influence neutrophil phenotype and contribute to epithelium proliferation. Conversely, during IBD, the epithelial barrier of the intestines is damaged, which allows for the entry of microbes into the tissue. Subsequently, surrounding macrophages predominantly express the M1 macrophage phenotype and release pro-inflammatory factors involved in neutrophil recruitment. Neutrophils arrive at the site of injury and proceed to produce anti-microbial factors to control infection; however, they may also further contribute to tissue destruction. Neutrophil accumulation in the tissue can also occur due to impairment in neutrophil apoptosis. *MIP-2* macrophage inflammatory protein 2; *MPO* myeloperoxidase; *ROS* reactive oxygen species; *TGF-β* transforming growth factor; *VEGF* vascular endothelial growth factor. (Stock images sourced from Servier Medical Art; Creative Commons)

The intestine is the most densely populated organ in the human body, containing over 10^12^ microorganisms that outnumber host cells by approximately 10:1 (Garrett et al. 2010). The structure of the gut consists of specialized intestinal cells that act as a physical barrier to separate the luminal content from the host tissues. Despite this, one of the requirements of the intestinal lining is to be highly permeable in order to regulate nutrient exchange during digestion. As a result, the microbial communities located in the mucosal region between the intestinal cells and lumen come in constant contact with the intestinal immune system (Mowat and Agace 2014). The mucosal microbiota coevolves with the intestinal immune system, thereby creating a unique microenvironment (Belkaid and Hand 2014). The immune population in the gut predominantly consists of monocytes and tissue-resident macrophages, which are concentrated in the lamina propria (Fig. 3) (Bain and Mowat 2014). Intestinal macrophages play a large role in maintaining intestinal homeostasis as they act as the first line of defense against any microorganisms that breach the intestinal lining (Belkaid and Hand 2014). Upon a microbial attack, a typical host immune response involves the activation of tissue-resident macrophages, which leads to the secretion of chemokines such as IL-8 that facilitate the recruitment of neutrophils to the gut. These neutrophils originate from the bloodstream and transmigrate across the vascular endothelium into the lamina propria (Beck-Schimmer et al. 2005; de Oliveira et al. 2013; Fournier and Parkos 2012). It is here where neutrophils perform antimicrobial functions and prevent any severe infections from occurring.

It is expected that the immune cells located in the intestines would be constantly activated, as they are in perpetual contact with the microbiota. However, studies have shown that these intestinal immune cells are capable of remaining in a state of anergy without compromising their functional capabilities (Smythies et al. 2005, 2010). In extra-gastrointestinal tissue, macrophages recognize and bind to moieties located on pathogens, leading to their activation. Recent evidence has shown that intestinal macrophages become partial to an immunosuppressive M2 profile in order to adjust to the local environment and prevent excessive inflammation. It was reported that the intestinal epithelium constitutively expresses TGF-β1, which could explain the preference for the anti-inflammatory M2 macrophages in the intestines rather than one that is of the M1 prolife (Avery et al. 1993). In fact, the M2 intestinal macrophages become tolerant to immunogenic peptides via the downregulation of pattern recognition receptors and constitutively produce IL-10 as well as TGF-β (Bain et al. 2013; Fadok et al. 1998; Smith et al. 2001). Both IL-10 and TGF-β have been shown to elicit anti-inflammatory effects on neutrophils, as these cytokines are able to regulate neutrophil recruitment, function and lifespan (Krause et al. 2015; Lewkowicz et al. 2006). Moreover, these intestinal macrophages play a large role in the clearance of neutrophils in the colon following inflammation as they release soluble Fas-ligand that binds to and causes neutrophil apoptosis (Fig. 3) (Duffield 2003).

The microenvironment in the gut is ever-changing due to our diet and lifestyle, thus the intestinal immune system must constantly adapt to maintain homeostasis. A disruption in the intestinal immune system has been shown to have both local and systemic effects, such that changes in the gastrointestinal environment lead to the onset of various diseases that are not confined to the gut (Ho et al. 2015). Locally, a variety of infections in the gut can occur and, due to the exponential growth of microbes, it is essential that the immune response is potent and quick. Neutrophils and macrophages have been shown to cooperate in order to enhance the immune response during infectious diseases. In one study, zebrafish were infected with *Mycobacterium marinum* to mimic chronic granulomatous disease, which primarily affects the gastrointestinal tract. It was reported that neutrophils were recruited to granulomas by dying macrophages, which they then internalize and destroy (Yang et al. 2012). *Shigella flexneri*, a pathogen that causes dysentery, is rapidly phagocytosed by macrophages and neutrophils upon infection. Macrophages that internalized the bacteria quickly died. Conversely, neutrophils infected with *S. flexneri* successfully degraded the bacteria and proceeded to engulf the dead macrophages (Mostowy et al. 2013). *Clostridium difficile* and *Staphylococcus aureus* are both members of the host intestinal microbiota of most individuals. Although both neutrophils and macrophages work together to overcome infection by these bacteria when they overgrow, the outcome is not the same. During infection with *C. difficile*, neutrophil recruitment is enhanced by MIP-2 released by macrophages (Castagliuolo et al. 1998). On the other hand, *S. aureus* exploits this relationship to aid in its own survival by hindering the uptake of apoptotic neutrophils by macrophages, resulting in necrosis (Greenlee-Wacker et al. 2014). Therefore, the interaction between neutrophils and macrophages in the gut is clearly essential against microbial invasions, though it may also be exploited in some infections to the benefit of the bacteria.

In addition to constant microbial exposure, the gut is also prone to autoimmune diseases: inflammatory bowel disease (IBD), of which the most common forms are Crohn’s disease (CD) and ulcerative colitis (UC). CD is characterized by widespread inflammation throughout the gastrointestinal tract whereas UC is confined to the large intestine, more specifically the mucosa (Xavier and Podolsky 2007). IBD in humans is driven by T cells due to their pivotal role against pathogens; however, there is also the involvement of immune cells such as neutrophils and macrophages during disease as shown by their accumulation in the inflamed mucosa (Larmonier et al. 2015). In order to study this involvement, various animal models have been adopted to replicate the symptoms of CD and UC in humans. These include the trinitrobenzene sulfonic acid (TNBS), dextran sulfate sodium (DSS) and adopted T cell transfer models (Mizoguchi 2012). These models are commonly used to evaluate the efficacy of therapeutics; however, it is important to consider that, although the pathologies induced by the animal models are not identical to that of human IBD, they are designed to mimic aspects of the human disease.

The role of macrophages and neutrophils differ between CD and UC. It has been noted that there are high levels of pro-inflammatory macrophages detected in the gastrointestinal tissue of CD patients (Thiesen et al. 2014). In contrast, neutrophil accumulation predominates in UC patients (Harbord et al. 2006; Marks et al. 2006). It was reported that cultured macrophages from CD patients secreted less chemotactic mediators such as IL-8 as well as impaired pro-inflammatory cytokine production following stimulation with *Escherichia coli* (Marks et al. 2006; Smith et al. 2009). Conversely, UC is thought to be a neutrophil-driven disease (Bressenot et al. 2015). Indeed, neutrophils contribute to disease pathology via the excessive production of pro-inflammatory factors such as MPO and ROS, leading to tissue damage (Fig. 3) (Wéra et al. 2016). During DSS-induced colitis, mice deficient of macrophages resulted in exacerbated disease pathology, higher levels of *CXCL1* expression and MPO activity compared to wild-type mice. Not surprisingly, there was a concurrent increase in the number of neutrophils in the colon. Subsequently, neutrophil depletion prevented the increase in disease severity in these mice (Qualls et al. 2006). The findings from this study suggest that gastrointestinal macrophages have a protective effect during colitis by downregulating neutrophil recruitment as well as their activity. Interestingly, recent studies have shown that neutrophils are also able to play a protective role during TNBS- and DSS-induced colitis (Kühl et al. 2007; Rong et al. 2011). The discrepancy in results from the aforementioned studies could be due to a change in the inflammatory environment of the colon as it is dependent on the model of colitis, species used and the setup of the study.

One of the characteristics of IBD is an inflamed and damaged mucosa (Fig. 3). Macrophages play a major role in wound healing and mucosal repair by the release of reparative factors, such as vascular endothelial growth factor (VEGF) and TGF-β (Brancato and Albina 2011). On the other hand, neutrophils secrete a variety of tissue damaging factors, such as oxidants and granulocytic proteins, during the immune response and may negatively influence wound repair (Koh and DiPietro 2011 Wilgus et al. 2003). Thus, the role of macrophages in inducing apoptosis and clearing neutrophils from inflamed gut tissues are essential in preventing intestinal tissue necrosis and pathology. Indeed, apoptotic intestinal epithelial cells were observed to be abundant in patients with IBD. In contrast, there is evidence of delayed apoptosis in immune cells such as T cells and neutrophils during IBD (Brannigan et al. 2000; Souza et al. 2005). The cell death pathway of neutrophils from IBD patients appeared to be delayed as there was a decrease in the expression of pro-caspase 3. Furthermore, the high levels of IL-8 found in the serum of IBD patients compared to healthy controls may explain the increased resistance to cell death by the Fas-ligand produced by macrophages (Brannigan et al. 2000). It is conceivable that this impairment of apoptotic machinery could account for the aforementioned accumulation of leukocytes in the colon during colitis. This study highlights the fact that neutrophil apoptosis is delayed during IBD; however, further studies are warranted to determine whether it occurs before, during or after the onset of disease. While neutrophils have been shown to be capable of producing proangiogenic and reparative VEGF, as well as expressing IL-10 that could potentially modulate both monocyte and macrophage polarization to pro-reparative phenotypes, it is currently unclear whether neutrophils promote these phenotypic changes in the inflamed colon. Additionally, it is unknown whether the delayed neutrophil apoptosis in IBD is mediated by impaired phagocytic function in macrophages in the gut. The discovery of other mechanisms that contribute to impaired neutrophil apoptosis will be important for the development of effective interventions and therapeutics in the future.

## Conclusions

There have been significant discoveries in our understanding of the interaction between neutrophils and monocytes/macrophages. This immunological relationship has evolved in such a way that it is dynamic and able to adapt to various pathogens and microenvironment settings. However, it should be noted that the neutrophil and monocyte/macrophage interaction may not always be beneficial to the host. As discussed in this review, both of these cells have been demonstrated to be capable of driving various autoimmune and inflammatory diseases. Nonetheless, depletion studies and the use of novel transgenic animal models in recent years have begun to reveal key mechanisms and identify critical roles of neutrophils and monocytes/macrophages during an aberrant immune response. Clearly, a better understanding of the relationship shared between neutrophils and monocytes/macrophages and their complex interactions, is critical for the development of therapeutics for numerous diseases in the future.

## References

[CR1] Abel RM, Buckley M, Austen W, Barnett G, Beck Jr C, Fischer J (1976). Etiology, incidence, and prognosis of renal failure following cardiac operations. Results of a prospective analysis of 500 consecutive patients. J Thorac Cardiovasc Surg.

[CR2] Adamson S, Leitinger N (2011). Phenotypic modulation of macrophages in response to plaque lipids. Curr Opin Lipidol.

[CR3] Agerberth B, Charo J, Werr J, Olsson B, Idali F, Lindbom L, Kiessling R, Jörnvall H, Wigzell H, Gudmundsson GH (2000). The human antimicrobial and chemotactic peptides LL-37 and α-defensins are expressed by specific lymphocyte and monocyte populations. Blood.

[CR4] Akcay A, Nguyen Q, Edelstein CL (2010) Mediators of inflammation in acute kidney injury. Mediat Inflamm 2009 10.1155/2009/137072 PMC2825552 20182538

[CR5] Alberts B, Johnson A, Lewis J, Raff M, Roberts K, Walter P (2002). Molecular biology of the cell.

[CR6] Allam R, Scherbaum CR, Darisipudi MN, Mulay SR, Hägele H, Lichtnekert J, Hagemann JH, Rupanagudi KV, Ryu M, Schwarzenberger C (2012). Histones from dying renal cells aggravate kidney injury via TLR2 and TLR4. J Am Soc Nephrol.

[CR7] Allenbach C, Zufferey C, Perez C, Launois P, Mueller C, Tacchini-Cottier F (2006). Macrophages induce Neutrophil apoptosis through membrane TNF, a process amplified by *Leishmania major*. J Immunol.

[CR8] Andzinski L, C-F W, Lienenklaus S, Kröger A, Weiss S, Jablonska J (2015). Delayed apoptosis of tumor associated neutrophils in the absence of endogenous IFN-β. Int J Cancer.

[CR9] Avery A, Paraskeva C, Hall P, Flanders KC, Sporn M, Moorghen M (1993). TGF-beta expression in the human colon: differential immunostaining along crypt epithelium. Br J Cancer.

[CR10] Bain CC, Mowat AM (2014). Macrophages in intestinal homeostasis and inflammation. Immunol Rev.

[CR11] Bain CC, Scott CL, Uronen-Hansson H, Gudjonsson S, Jansson O, Grip O, Guilliams M, Malissen B, Agace WW, Mowat AM (2013). Resident and pro-inflammatory macrophages in the colon represent alternative context-dependent fates of the same Ly6Chi monocyte precursors. Mucosal Immunol.

[CR12] Barnathan ES, Raghunath P, Tomaszewski JE, Ganz T, Cines DB (1997). Immunohistochemical localization of defensin in human coronary vessels. Am J Pathol.

[CR13] Barry KC, Fontana MF, Portman JL, Dugan AS, Vance RE (2013) Interleukin-1α signaling initiates the inflammatory response to virulent Legionella pneumophila in vivo. J Immunol (Baltimore, Md : 1950) 190:6329-6339 10.4049/jimmunol.1300100 PMC3682686 23686480

[CR14] Baud L, Ardaillou R (1993). Involvement of reactive oxygen species in kidney damage. Br Med Bull.

[CR15] Beck-Schimmer B, Schwendener R, Pasch T, Reyes L, Booy C, Schimmer RC (2005). Alveolar macrophages regulate neutrophil recruitment in endotoxin-induced lung injury. Respir Res.

[CR16] Becquet O, Pasche J, Gatti H, Chenel C, Abély M, Morville P, Pietrement C (2010). Acute post-streptococcal glomerulonephritis in children of French Polynesia: a 3-year retrospective study. Pediatr Nephrol.

[CR17] Belkaid Y, Hand Timothy W (2014). Role of the microbiota in immunity and inflammation. Cell.

[CR18] Beyrau M, Bodkin JV, Nourshargh S (2012). Neutrophil heterogeneity in health and disease: a revitalized avenue in inflammation and immunity. Open Biology.

[CR19] Bots ML, Hoes AW, Koudstaal PJ, Hofman A, Grobbee DE (1997). Common carotid intima-media thickness and risk of stroke and myocardial infarction. Circulation.

[CR20] Braehler S, Cheung M, Huang D, Akers W, Kim A (2016) THU0244 Noninvasive Assessment of Macrophage Activation in Experimental Glomerulonephritis Using Optical Imaging with Near-Infrared Light Serves as A Surrogate of Disease Onset. BMJ Publishing

[CR21] Brancato SK, Albina JE (2011). Wound macrophages as key regulators of repair: origin, phenotype, and function. Am J Pathol.

[CR22] Brannigan AEOCPR, Hurley H, O’Neill A, Brady HRFJM, Watson RW (2000). Neutrophil apoptosis is delayed in patients with inflammatory bowel disease. Shock.

[CR23] Bressenot A, Salleron J, Bastien C, Danese S, Boulagnon-Rombi C, Peyrin-Biroulet L (2015). Comparing histological activity indexes in UC. Gut.

[CR24] Butterfield TA, Best TM, Merrick MA (2006). The dual roles of Neutrophils and macrophages in inflammation: a critical balance between tissue damage and repair. J Athl Train.

[CR25] Cantürk NZ, Esen N, Vural B, Cantürk Z, Kirkali G, Oktay G, Solakoglu S (2001). The relationship between Neutrophils and Incisional wound healing. Skin Pharmacol Physiol.

[CR26] Castagliuolo I, Keates AC, Wang CC, Pasha A, Valenick L, Kelly CP, Nikulasson ST, LaMont JT, Pothoulakis C (1998). *Clostridium difficile* toxin a stimulates macrophage- inflammatory Protein-2 production in rat intestinal epithelial cells. J Immunol.

[CR27] Chen F, Wu W, Millman A, Craft JF, Chen E, Patel N, Boucher JL, Urban JF, Kim CC, Gause WC (2014). Neutrophils prime a long-lived effector macrophage phenotype that mediates accelerated helminth expulsion. Nat Immunol.

[CR28] Chertov O, Ueda H, LL X, Tani K, Murphy WJ, Wang JM, Howard OMZ, Sayers TJ, Oppenheim JJ (1997). Identification of human Neutrophil-derived Cathepsin G and Azurocidin/CAP37 as Chemoattractants for mononuclear cells and Neutrophils. J Exp Med.

[CR29] Chertov O, Ueda H, LL X, Tani K, Murphy WJ, Wang JM, Howard OZ, Sayers TJ, Oppenheim JJ (1997). Identification of human neutrophil-derived cathepsin G and azurocidin/CAP37 as chemoattractants for mononuclear cells and neutrophils. J Exp Med.

[CR30] Ciornei CD, Tapper H, Bjartell A, Sternby NH, Bodelsson M (2006). Human antimicrobial peptide LL-37 is present in atherosclerotic plaques and induces death of vascular smooth muscle cells: a laboratory study. BMC Cardiovasc Disord.

[CR31] Cuartero MI, Ballesteros I, Moraga A, Nombela F, Vivancos J, Hamilton JA, Corbí ÁL, Lizasoain I, Moro MA (2013). N2 Neutrophils, novel players in brain inflammation after stroke. Modulation by the PPARγ Agonist Rosiglitazone. Stroke.

[CR32] Daley JM, Thomay AA, Connolly MD, Reichner JS, Albina JE (2008). Use of Ly6G-specific monoclonal antibody to deplete neutrophils in mice. J Leukoc Biol.

[CR33] Davies LC, Taylor PR (2015). Tissue-resident macrophages: then and now. Immunology.

[CR34] De Filippo K, Henderson RB, Laschinger M, Hogg N (2008). Neutrophil chemokines KC and macrophage-inflammatory Protein-2 are newly synthesized by tissue macrophages using distinct TLR Signaling pathways. J Immunol.

[CR35] de Oliveira S, Reyes-Aldasoro CC, Candel S, Renshaw SA, Mulero V, Calado Â (2013). Cxcl8 (Interleukin-8) mediates neutrophil recruitment and behavior in the zebrafish inflammatory response. J Immunol (Baltimore, Md: 1950).

[CR36] de Oliveira S, Rosowski EE, Huttenlocher A (2016). Neutrophil migration in infection and wound repair: going forward in reverse. Nat Rev Immunol.

[CR37] Devi S, Li A, Westhorpe CL, Lo CY, Abeynaike LD, Snelgrove SL, Hall P, Ooi JD, Sobey CG, Kitching AR (2013). Multiphoton imaging reveals a new leukocyte recruitment paradigm in the glomerulus. Nat Med.

[CR38] Drechsler M, Megens RT, van Zandvoort M, Weber C, Soehnlein O (2010). Hyperlipidemia-triggered neutrophilia promotes early atherosclerosis. Circulation.

[CR39] Duffield JS (2003). The inflammatory macrophage: a story of Jekyll and Hyde. Clin Sci.

[CR40] Elmore S (2007). Apoptosis: a review of programmed cell death. Toxicol Pathol.

[CR41] Fadok VA, Bratton DL, Konowal A, Freed PW, Westcott JY, Henson PM (1998). Macrophages that have ingested apoptotic cells in vitro inhibit proinflammatory cytokine production through autocrine/paracrine mechanisms involving TGF-beta, PGE2, and PAF. J Clin Investig.

[CR42] Ferrario F, Castiglione A, Colasanti G, di Belgioioso GB, Bertoli S, D’Amico G, Nava S (1985). The detection of monocytes in human glomerulonephritis. Kidney Int.

[CR43] Finsterbusch M, Hall P, Li A, Devi S, Westhorpe CL, Kitching AR, Hickey MJ (2016) Patrolling monocytes promote intravascular neutrophil activation and glomerular injury in the acutely inflamed glomerulus. Proc Natl Acad Sci U S A 201606253 10.1073/pnas.1606253113 PMC5024581 27528685

[CR44] Fournier BM, Parkos CA (2012). The role of neutrophils during intestinal inflammation. Mucosal Immunol.

[CR45] Fridlender ZG, Sun J, Kim S, Kapoor V, Cheng G, Ling L, Worthen GS, Albelda SM (2009). Polarization of tumor-associated Neutrophil (TAN) phenotype by TGF-β: “N1” versus “N2” TAN. Cancer Cell.

[CR46] Fuchs TA, Abed U, Goosmann C, Hurwitz R, Schulze I, Wahn V, Weinrauch Y, Brinkmann V, Zychlinsky A (2007). Novel cell death program leads to neutrophil extracellular traps. J Cell Biol.

[CR47] Garrett WS, Gordon JI, Glimcher LH (2010). Homeostasis and inflammation in the intestine. Cell.

[CR48] Gautam N, Maria Olofsson A, Herwald H, Iversen LF, Lundgren-Akerlund E, Hedqvist P, Arfors K-E, Flodgaard H, Lindbom L (2001). Heparin-binding protein (HBP/CAP37): a missing link in neutrophil-evoked alteration of vascular permeability. Nat Med.

[CR49] Geissmann F, Manz MG, Jung S, Sieweke MH, Merad M, Ley K (2010). Development of monocytes, macrophages and dendritic cells. Science.

[CR50] Gerrity RG (1981). The role of the monocyte in atherogenesis: I. Transition of blood-borne monocytes into foam cells in fatty lesions. Am J Pathol.

[CR51] Grau AJ, Weimar C, Buggle F, Heinrich A, Goertler M, Neumaier S, Glahn J, Brandt T, Hacke W, Diener H-C (2001). Risk factors, outcome, and treatment in subtypes of ischemic stroke. Stroke.

[CR52] Greenlee-Wacker MC, Rigby KM, Kobayashi SD, Porter AR, DeLeo FR, Nauseef WM (2014) Phagocytosis of *Staphylococcus aureus* by human neutrophils prevents macrophage efferocytosis and induces programmed necrosis. J Immunol (Baltimore, Md : 1950) 192:4709-4717 10.4049/jimmunol.1302692 PMC4011196 24729616

[CR53] Hamilton TA, Zhao C, Pavicic PG, Datta S (2014). Myeloid Colony-stimulating factors as regulators of macrophage polarization. Front Immunol.

[CR54] Hamouda M, Mrabet I, Dhia NB, Aloui S, Letaif A, Frih M, Skhiri H, Elmay M (2014). Acute post-infectious glomerulonephritis in adults: a single center report. Saudi J Kidney Dis Transpl.

[CR55] Harbord MWN, Marks DJB, Forbes A, Bloom SL, Day RM, Segal AW (2006). Impaired neutrophil chemotaxis in Crohn’s disease relates to reduced production of chemokines and can be augmented by granulocyte-colony stimulating factor. Aliment Pharmacol Ther.

[CR56] Ho JTK, Chan GCF, Li JCB (2015). Systemic effects of gut microbiota and its relationship with disease and modulation. BMC Immunol.

[CR57] Hooke DH, Gee DC, Atkins RC (1987). Leukocyte analysis using monoclonal antibodies in human glomerulonephritis. Kidney Int.

[CR58] Hu X, Chakravarty SD, Ivashkiv LB (2008). Regulation of IFN and TLR Signaling during macrophage activation by opposing feedforward and feedback inhibition mechanisms. Immunol Rev.

[CR59] Huang Z-S, Chien K-L, Yang C-Y, Tsai K-S, Wang C-H (2001). Peripheral differential leukocyte counts in humans vary with hyperlipidemia, smoking, and body mass index. Lipids.

[CR60] Ionita MG, van den Borne P, Catanzariti LM, Moll FL, de Vries J-PP, Pasterkamp G, Vink A, de Kleijn DP (2010). High neutrophil numbers in human carotid atherosclerotic plaques are associated with characteristics of rupture-prone lesions. Arterioscler Thromb Vasc Biol.

[CR61] Janeway CJ, Travers P, Walport M, Shlomchik M (2001). Immunobiology.

[CR62] Jang HR, Rabb H (2009). The innate immune response in ischemic acute kidney injury. Clin Immunol.

[CR63] Jegatheesan D, Nath K, Reyaldeen R, Sivasuthan G, John GT, Francis L, Rajmokan M, Ranganathan D (2016). Epidemiology of biopsy-proven glomerulonephritis in Queensland adults. Nephrology.

[CR64] Kanjanabuch T, Kittikowit W, Eiam-Ong S (2009). An update on acute postinfectious glomerulonephritis worldwide. Nature reviews. Nephrology.

[CR65] Karasawa K, Asano K, Moriyama S, Ushiki M, Monya M, Iida M, Kuboki E, Yagita H, Uchida K, Nitta K (2014) Vascular-resident CD169-positive monocytes and macrophages control neutrophil accumulation in the kidney with ischemia-reperfusion injury. J Am Soc Nephrol 2014020195 10.1681/ASN.2014020195 PMC4378108 25266072

[CR66] Kaushal GP, Basnakian AG, Shah SV (2004). Apoptotic pathways in ischemic acute renal failure. Kidney Int.

[CR67] Knight JS, Luo W, O’dell AA, Yalavarthi S, Zhao W, Subramanian V, Guo C, Grenn RC, Thompson PR, Eitzman DT (2014) Peptidylarginine deiminase inhibition reduces vascular damage and modulates innate immune responses in murine models of atherosclerosis. Circulation Research CIRCRESAHA 113(303312) 10.1161/CIRCRESAHA.114.303312 PMC4185401 24425713

[CR68] Koh TJ, DiPietro LA (2011). Inflammation and wound healing: the role of the macrophage. Expert Rev Mol Med.

[CR69] Krause P, Morris V, Greenbaum JA, Park Y, Bjoerheden U, Mikulski Z, Muffley T, Shui J-W, Kim G, Cheroutre H, Liu Y-C, Peters B, Kronenberg M, Murai M (2015) IL-10-producing intestinal macrophages prevent excessive antibacterial innate immunity by limiting IL-23 synthesis. Nature Commun 6:7055 10.1038/ncomms8055 PMC4428691 25959063

[CR70] Kühl AA, Kakirman H, Janotta M, Dreher S, Cremer P, Pawlowski NN, Loddenkemper C, Heimesaat MM, Grollich K, Zeitz M, Farkas S, Hoffmann JC (2007). Aggravation of different types of experimental colitis by depletion or adhesion blockade of Neutrophils. Gastroenterology.

[CR71] Kuligowski MP, Kwan RY, Lo C, Wong C, James WG, Bourges D, Ooi JD, Abeynaike LD, Hall P, Kitching AR (2009). Antimyeloperoxidase antibodies rapidly induce α4-integrin–dependent glomerular neutrophil adhesion. Blood.

[CR72] Larmonier CB, Shehab KW, Ghishan FK, Kiela PR (2015). T lymphocyte dynamics in inflammatory bowel diseases: role of the microbiome. Biomed Res Int.

[CR73] Lech M, Gröbmayr R, Ryu M, Lorenz G, Hartter I, Mulay SR, Susanti HE, Kobayashi KS, Flavell RA, Anders H-J (2014). Macrophage phenotype controls long-term AKI outcomes—kidney regeneration versus atrophy. J Am Soc Nephrol.

[CR74] Lee TD, Gonzalez ML, Kumar P, Chary-Reddy S, Grammas P, Pereira HA (2002). CAP37, a novel inflammatory mediator: its expression in endothelial cells and localization to atherosclerotic lesions. Am J Pathol.

[CR75] Lee TD, Gonzalez ML, Kumar P, Grammas P, Pereira HA (2003). CAP37, a neutrophil-derived inflammatory mediator, augments leukocyte adhesion to endothelial monolayers. Microvasc Res.

[CR76] Lee S, Huen S, Nishio H, Nishio S, Lee HK, Choi B-S, Ruhrberg C, Cantley LG (2011). Distinct macrophage phenotypes contribute to kidney injury and repair. J Am Soc Nephrol.

[CR77] Lee HY, Kim SD, Baek S-H, Choi JH, Bae Y-S (2013). Role of formyl peptide receptor 2 on the serum amyloid A-induced macrophage foam cell formation. Biochem Biophys Res Commun.

[CR78] Lee HY, Oh E, Kim SD, Seo JK, Bae Y-S (2014). Oxidized low-density lipoprotein-induced foam cell formation is mediated by formyl peptide receptor 2. Biochem Biophys Res Commun.

[CR79] Legedz L, Randon J, Sessa C, Baguet J-P, Feugier P, Cerutti C, McGregor J, Bricca G (2004). Cathepsin G is associated with atheroma formation in human carotid artery. J Hypertens.

[CR80] Lewkowicz P, Lewkowicz N, Sasiak A, Tchórzewski H (2006). Lipopolysaccharide-activated CD4+CD25+ T regulatory cells inhibit Neutrophil function and promote their apoptosis and death. J Immunol.

[CR81] Li S, Krawczeski CD, Zappitelli M, Devarajan P, Thiessen-Philbrook H, Coca SG, Kim RW, Parikh CR (2011). Incidence, risk factors, and outcomes of acute kidney injury after pediatric cardiac surgery–a prospective multicenter study. Crit Care Med.

[CR82] Li Z, Kang Z, Duan C, Wu T, Zhang L, Xun M, Ding Y, Zhang Y, Yin Y (2014). Clinical and pathological features of acute kidney injury in children. Ren Fail.

[CR83] Liu C, Li Y, Yu J, Feng L, Hou S, Liu Y, Guo M, Xie Y, Meng J, Zhang H, Xiao B, Ma C (2013). Targeting the Shift from M1 to M2 macrophages in experimental autoimmune encephalomyelitis mice treated with Fasudil. PLoS ONE.

[CR84] Ma Y, Yabluchanskiy A, Iyer RP, Cannon PL, Flynn ER, Jung M, Henry J, Cates CA, Deleon-Pennell KY, Lindsey ML (2016). Temporal neutrophil polarization following myocardial infarction. Cardiovasc Res.

[CR85] Mantovani A, Cassatella MA, Costantini C, Jaillon S (2011). Neutrophils in the activation and regulation of innate and adaptive immunity. Nat Rev Immunol.

[CR86] Marks DJB, Harbord MWN, MacAllister R, Rahman FZ, Young J, Al-Lazikani B, Lees W, Novelli M, Bloom S, Segal AW (2006). Defective acute inflammation in Crohn’s disease: a clinical investigation. Lancet.

[CR87] McCracken JM, Allen L-AH (2014). Regulation of human Neutrophil apoptosis and lifespan in health and disease. J Cell Death.

[CR88] Megens RT, Vijayan S, Lievens D, Doering Y, van Zandvoort MA, Grommes J, Weber C, Soehnlein O (2012). Presence of luminal neutrophil extracellular traps in atherosclerosis. Thromb Haemost.

[CR89] Mestas J, Ley K (2008). Monocyte-endothelial cell interactions in the development of atherosclerosis. Trends Cardiovasc Med.

[CR90] Mizoguchi A (2012) Animal Models of Inflammatory Bowel Disease. In: Conn PM (ed) Progress in Molecular Biology and Translational Science, vol 105. Academic, New York, pp 263–320 10.1016/B978-0-12-394596-9.00009-3 22137435

[CR91] Mohty D, Pibarot P, Després J-P, Côté C, Arsenault B, Cartier A, Cosnay P, Couture C, Mathieu P (2008). Association between plasma LDL particle size, valvular accumulation of oxidized LDL, and inflammation in patients with aortic stenosis. Arterioscler Thromb Vasc Biol.

[CR92] Mokart D, Kipnis E, Guerre-Berthelot P, Vey N, Capo C, Sannini A, Brun J-P, Blache J-L, Mege J-L, Blaise D, Guery BP (2008). Monocyte deactivation in neutropenic acute respiratory distress syndrome patients treated with granulocyte colony-stimulating factor. Crit Care.

[CR93] Mostowy S, Boucontet L, Mazon Moya MJ, Sirianni A, Boudinot P, Hollinshead M, Cossart P, Herbomel P, Levraud J-P, Colucci-Guyon E (2013). The Zebrafish as a new model for the in vivo study of Shigella flexneri interaction with phagocytes and bacterial Autophagy. PLoS Pathog.

[CR94] Mowat AM, Agace WW (2014). Regional specialization within the intestinal immune system. Nat Rev Immunol.

[CR95] Nahrendorf M, Swirski FK (2015). Neutrophil-macrophage communication in inflammation and atherosclerosis. Science.

[CR96] Nakazawa D, Shida H, Kusunoki Y, Miyoshi A, Nishio S, Tomaru U, Atsumi T, Ishizu A (2016). The responses of macrophages in interaction with neutrophils that undergo NETosis. J Autoimmun.

[CR97] Nauseef WM (2007). How human neutrophils kill and degrade microbes: an integrated view. Immunol Rev.

[CR98] O’Sullivan KM, Lo CY, Summers SA, Elgass KD, McMillan PJ, Longano A, Ford SL, Gan P-Y, Kerr PG, Kitching AR (2015). Renal participation of myeloperoxidase in antineutrophil cytoplasmic antibody (ANCA)-associated glomerulonephritis. Kidney Int.

[CR99] Odobasic D, Kitching AR, Semple TJ, Holdsworth SR (2007). Endogenous myeloperoxidase promotes neutrophil-mediated renal injury, but attenuates T cell immunity inducing crescentic glomerulonephritis. J Am Soc Nephrol.

[CR100] Okpechi I, Swanepoel C, Duffield M, Mahala B, Wearne N, Alagbe S, Barday Z, Arendse C, Rayner B (2010). Patterns of renal disease in cape town South Africa: a 10-year review of a single-centre renal biopsy database. Nephrology dialysis. Transplantation.

[CR101] O’Leary DH, Polak JF, Kronmal RA, Manolio TA, Burke GL, Wolfson Jr SK (1999). Carotid-artery intima and media thickness as a risk factor for myocardial infarction and stroke in older adults. N Engl J Med.

[CR102] Østerud B, Bjørklid E (2003). Role of monocytes in atherogenesis. Physiol Rev.

[CR103] Påhlman LI, Mörgelin M, Eckert J, Johansson L, Russell W, Riesbeck K, Soehnlein O, Lindbom L, Norrby-Teglund A, Schumann RR, Björck L, Herwald H (2006). Streptococcal M protein: a multipotent and powerful inducer of inflammation. J Immunol.

[CR104] Podrez EA, Schmitt D, Hoff HF, Hazen SL (1999). Myeloperoxidase-generated reactive nitrogen species convert LDL into an atherogenic form in vitro. J Clin Investig.

[CR105] Poon IKH, Lucas CD, Rossi AG, Ravichandran KS (2014). Apoptotic cell clearance: basic biology and therapeutic potential. Nat Rev Immunol.

[CR106] Qin Y, Fan F, Zhao Y, Cui Y, Wei X, Kohama K, Gordon JR, Li F, Gao Y (2013). Recombinant human CXCL8 (3-72) K11R/G31P regulates smooth muscle cell proliferation and migration through blockage of interleukin-8 receptor. IUBMB Life.

[CR107] Qualls JE, Kaplan AM, van Rooijen N, Cohen DA (2006). Suppression of experimental colitis by intestinal mononuclear phagocytes. J Leukoc Biol.

[CR108] Ramanathan G, Abeyaratne A, Sundaram M, Fernandes DK, Pawar B, Perry GJ, Sajiv C, Majoni SW (2017). Analysis of clinical presentation, pathological spectra, treatment and outcomes of biopsy-proven acute postinfectious glomerulonephritis in adult indigenous people of the northern territory of Australia. Nephrology.

[CR109] Rao X, Zhong J, Sun Q (2014). The heterogenic properties of monocytes/macrophages and neutrophils in inflammatory response in diabetes. Life Sci.

[CR110] Remijsen Q, Kuijpers T, Wirawan E, Lippens S, Vandenabeele P, Berghe TV (2011). Dying for a cause: NETosis, mechanisms behind an antimicrobial cell death modality. Cell Death Differ.

[CR111] Rong Z, Sachiko I, Naomi N, Zhao C, Haruhiko S, Ken-ichi I (2011). Up-regulation of Gr1+CD11b+ population in spleen of Dextran Sulfate sodium administered mice works to repair colitis. Inflammation & Allergy - Drug Targets (Discontinued).

[CR112] Rosner MH, Okusa MD (2006). Acute kidney injury associated with cardiac surgery. Clin J Am Soc Nephrol.

[CR113] Rotzius P, Thams S, Soehnlein O, Kenne E, Tseng C-N, Björkström NK, Malmberg K-J, Lindbom L, Eriksson EE (2010). Distinct infiltration of neutrophils in lesion shoulders in ApoE−/− mice. Am J Pathol.

[CR114] Scannell M, Flanagan MB, deStefani A, Wynne KJ, Cagney G, Godson C, Maderna P (2007). Annexin-1 and peptide derivatives are released by apoptotic cells and stimulate Phagocytosis of apoptotic Neutrophils by macrophages. J Immunol.

[CR115] Shankar A, Mitchell P, Rochtchina E, Wang JJ (2007). The association between circulating white blood cell count, triglyceride level and cardiovascular and all-cause mortality: population-based cohort study. Atherosclerosis.

[CR116] Shaul ME, Levy L, Sun J, Mishalian I, Singhal S, Kapoor V, Horng W, Fridlender G, Albelda SM, Fridlender ZG (2016). Tumor-associated neutrophils display a distinct N1 profile following TGFβ modulation: a transcriptomics analysis of pro- vs. antitumor TANs. OncoImmunology.

[CR117] Shiohara M, Gombart AF, Sekiguchi Y, Hidaka E, Ito S, Yamazaki T, Koeffler HP, Komiyama A (2004). Phenotypic and functional alterations of peripheral blood monocytes in neutrophil-specific granule deficiency. J Leukoc Biol.

[CR118] Silva MT (2010). When two is better than one: macrophages and neutrophils work in concert in innate immunity as complementary and cooperative partners of a myeloid phagocyte system. J Leukoc Biol.

[CR119] Silva MT, Correia-Neves M (2012). Neutrophils and macrophages: the main partners of phagocyte cell systems. Front Immunol.

[CR120] Silva MT, Silva MN, Appelberg R (1989). Neutrophil-macrophage cooperation in the host defence against mycobacterial infections. Microb Pathog.

[CR121] Smith PD, Smythies LE, Mosteller-Barnum M, Sibley DA, Russell MW, Merger M, Sellers MT, Orenstein JM, Shimada T, Graham MF, Kubagawa H (2001). Intestinal macrophages lack CD14 and CD89 and consequently are down-regulated for LPS- and IgA-mediated activities. J Immunol.

[CR122] Smith AM, Rahman FZ, Hayee BH, Graham SJ, Marks DJB, Sewell GW, Palmer CD, Wilde J, Foxwell BMJ, Gloger IS, Sweeting T, Marsh M, Walker AP, Bloom SL, Segal AW (2009). Disordered macrophage cytokine secretion underlies impaired acute inflammation and bacterial clearance in Crohn’s disease. J Exp Med.

[CR123] Smythies LE, Sellers M, Clements RH, Mosteller-Barnum M, Meng G, Benjamin WH, Orenstein JM, Smith PD (2005). Human intestinal macrophages display profound inflammatory anergy despite avid phagocytic and bacteriocidal activity. J Clin Investig.

[CR124] Smythies LE, Shen R, Bimczok D, Novak L, Clements RH, Eckhoff DE, Bouchard P, George MD, WK H, Dandekar S, Smith PD (2010). Inflammation Anergy in human intestinal macrophages is due to Smad-induced IκBα expression and NF-κB inactivation. J Biol Chem.

[CR125] Soehnlein O, Weber C (2009) Myeloid cells in atherosclerosis: initiators and decision shapers. Seminars in immunopathology, vol 31. Springer,Berlin, pp 35-47 10.1007/s00281-009-0141-z 19238385

[CR126] Soehnlein O, Xie X, Ulbrich H, Kenne E, Rotzius P, Flodgaard H, Eriksson EE, Lindbom L (2005). Neutrophil-derived heparin-binding protein (HBP/CAP37) deposited on endothelium enhances monocyte arrest under flow conditions. J Immunol.

[CR127] Soehnlein O, Kai-Larsen Y, Frithiof R, Sorensen OE, Kenne E, Scharffetter-Kochanek K, Eriksson EE, Herwald H, Agerberth B, Lindbom L (2008). Neutrophil primary granule proteins HBP and HNP1–3 boost bacterial phagocytosis by human and murine macrophages. J Clin Invest.

[CR128] Soehnlein O, Kenne E, Rotzius P, Eriksson E, Lindbom L (2008). Neutrophil secretion products regulate anti-bacterial activity in monocytes and macrophages. Clin Exp Immunol.

[CR129] Soehnlein O, Zernecke A, Eriksson EE, Rothfuchs AG, Pham CT, Herwald H, Bidzhekov K, Rottenberg ME, Weber C, Lindbom L (2008). Neutrophil secretion products pave the way for inflammatory monocytes. Blood.

[CR130] Soehnlein O, Weber C, Lindbom L (2009). Neutrophil granule proteins tune monocytic cell function. Trends Immunol.

[CR131] Soehnlein O, Zernecke A, Weber C (2009). Neutrophils launch monocyte extravasation by release of granule proteins. Thromb Haemost.

[CR132] Souza HSP, Tortori CJA, Castelo-Branco MTL, Carvalho ATP, Margallo VS, Delgado CFS, Dines I, Elia CCS (2005). Apoptosis in the intestinal mucosa of patients with inflammatory bowel disease: evidence of altered expression of FasL and perforin cytotoxic pathways. Int J Color Dis.

[CR133] Stark MA, Huo Y, Burcin TL, Morris MA, Olson TS, Ley K (2005). Phagocytosis of apoptotic Neutrophils regulates Granulopoiesis via IL-23 and IL-17. Immunity.

[CR134] Sunderkötter C, Nikolic T, Dillon MJ, van Rooijen N, Stehling M, Drevets DA, Leenen PJM (2004). Subpopulations of mouse blood Monocytes differ in maturation stage and inflammatory response. J Immunol.

[CR135] Takano T, Azuma N, Satoh M, Toda A, Hashida Y, Satoh R, Hohdatsu T (2009). Neutrophil survival factors (TNF-alpha, GM-CSF, and G-CSF) produced by macrophages in cats infected with feline infectious peritonitis virus contribute to the pathogenesis of granulomatous lesions. Arch Virol.

[CR136] Tan BH, Meinken C, Bastian M, Bruns H, Legaspi A, Ochoa MT, Krutzik SR, Bloom BR, Ganz T, Modlin RL, Stenger S (2006). Macrophages acquire Neutrophil granules for antimicrobial activity against intracellular pathogens. J Immunol.

[CR137] Tang T, Rosenkranz A, Assmann KJ, Goodman MJ, Gutierrez-Ramos J-C, Carroll MC, Cotran RS, Mayadas TN (1997). A role for Mac-1 (CDIIb/CD18) in immune complex–stimulated neutrophil function in vivo: Mac-1 deficiency abrogates sustained Fcγ receptor–dependent neutrophil adhesion and complement-dependent proteinuria in acute glomerulonephritis. J Exp Med.

[CR138] Thiesen S, Janciauskiene S, Uronen-Hansson H, Agace W, Högerkorp C-M, Spee P, Håkansson K, Grip O (2014). CD14(hi)HLA-DR(dim) macrophages, with a resemblance to classical blood monocytes, dominate inflamed mucosa in Crohn’s disease. J Leukoc Biol.

[CR139] Thurman JM, Ljubanovic D, Edelstein CL, Gilkeson GS, Holers VM (2003). Lack of a functional alternative complement pathway ameliorates ischemic acute renal failure in mice. J Immunol.

[CR140] Undurti A, Huang Y, Lupica JA, Smith JD, DiDonato JA, Hazen SL (2009). Modification of high density lipoprotein by myeloperoxidase generates a pro-inflammatory particle. J Biol Chem.

[CR141] van der Does AM, Beekhuizen H, Ravensbergen B, Vos T, Ottenhoff TH, van Dissel JT, Drijfhout JW, Hiemstra PS, Nibbering PH (2010). LL-37 directs macrophage differentiation toward macrophages with a proinflammatory signature. J Immunol.

[CR142] Wan M, van der Does AM, Tang X, Lindbom L, Agerberth B, Haeggström JZ (2014). Antimicrobial peptide LL-37 promotes bacterial phagocytosis by human macrophages. J Leukoc Biol.

[CR143] Warnatsch A, Ioannou M, Wang Q, Papayannopoulos V (2015). Neutrophil extracellular traps license macrophages for cytokine production in atherosclerosis. Science.

[CR144] Weber C, Noels H (2011). Atherosclerosis: current pathogenesis and therapeutic options. Nat Med.

[CR145] Weidner S, Carl M, Riess R, Rupprecht HD (2004). Histologic analysis of renal leukocyte infiltration in antineutrophil cytoplasmic antibody–associated vasculitis: importance of monocyte and neutrophil infiltration in tissue damage. Arthritis Rheumatol.

[CR146] Wéra O, Lancellotti P, Oury C (2016). The dual role of Neutrophils in inflammatory bowel diseases. J Clin Med.

[CR147] Wilgus TA, Vodovotz Y, Vittadini E, Clubbs EA, Oberyszyn TM (2003). Reduction of scar formation in full-thickness wounds with topical celecoxib treatment. Wound Repair Regen.

[CR148] Woollard KJ, Geissmann F (2010). Monocytes in atherosclerosis: subsets and functions. Nature reviews. Cardiology.

[CR149] Wylie J, Stubbs H (2009). The plague of Athens: 430–428 B.C. epidemic and Epizoötic. Class Q.

[CR150] Wynn TA, Vannella KM (2016). Macrophages in tissue repair, regeneration, and fibrosis. Immunity.

[CR151] Xavier RJ, Podolsky DK (2007). Unravelling the pathogenesis of inflammatory bowel disease. Nature.

[CR152] Xiao H, Heeringa P, Liu Z, Huugen D, Hu P, Maeda N, Falk RJ, Jennette JC (2005). The role of neutrophils in the induction of glomerulonephritis by anti-myeloperoxidase antibodies. Am J Pathol.

[CR153] Yamashiro S, Kamohara H, Wang J-M, Yang D, Gong W-H, Yoshimura T (2001). Phenotypic and functional change of cytokine-activated neutrophils: inflammatory neutrophils are heterogeneous and enhance adaptive immune responses. J Leukoc Biol.

[CR154] Yang D, Chen Q, Schmidt AP, Anderson GM, Wang JM, Wooters J, Oppenheim JJ, Chertov O (2000). LL-37, the neutrophil granule–and epithelial cell–derived cathelicidin, utilizes formyl peptide receptor–like 1 (FPRL1) as a receptor to chemoattract human peripheral blood neutrophils, monocytes, and T cells. J Exp Med.

[CR155] Yang C-T, Cambier CJ, Davis JM, Hall CJ, Crosier PS, Ramakrishnan L (2012). Neutrophils exert protection in the early Tuberculous Granuloma by oxidative killing of Mycobacteria Phagocytosed from infected macrophages. Cell Host Microbe.

[CR156] Zernecke A, Bot I, Djalali-Talab Y, Shagdarsuren E, Bidzhekov K, Meiler S, Krohn R, Schober A, Sperandio M, Soehnlein O (2008). Protective role of CXC receptor 4/CXC ligand 12 unveils the importance of neutrophils in atherosclerosis. Circ Res.

[CR157] Zhang WR, Garg AX, Coca SG, Devereaux PJ, Eikelboom J, Kavsak P, McArthur E, Thiessen-Philbrook H, Shortt C, Shlipak M (2015). Plasma IL-6 and IL-10 concentrations predict AKI and long-term mortality in adults after cardiac surgery. J Am Soc Nephrol.

[CR158] Zhou J, Stohlman SA, Hinton DR, Marten NW (2003). Neutrophils promote mononuclear cell infiltration during viral-induced encephalitis. J Immunol.

[CR159] Zughaier SM, Shafer WM, Stephens DS (2005). Antimicrobial peptides and endotoxin inhibit cytokine and nitric oxide release but amplify respiratory burst response in human and murine macrophages. Cell Microbiol.

